# Effect of hexavalent chromium on growth performance and metabolism in broiler chicken

**DOI:** 10.3389/fvets.2023.1273944

**Published:** 2023-09-26

**Authors:** Guorong Zhuo, Lei Wang, Muhammad Ali, Zheng Jing, Mohammad Farooque Hassan

**Affiliations:** ^1^College of Small Animal Science and Technology, Jiangsu Agri-Animal Husbandry Vocational College, Taizhou, China; ^2^College of Veterinary Medicine, Huazhong Agricultural University, Wuhan, China; ^3^Basic Health Unit, Department of Health, Dera Ghazi Khan, Pakistan; ^4^Shaheed Benazir Bhutto University of Veterinary & Animal Sciences, Sakrand, Pakistan

**Keywords:** hexavalent chromium Cr (VI), growth performance, serum metabolism, hazard, toxicity

## Abstract

Hexavalent chromium Cr (VI) is one of the most hazardous heavy metals in the environment and is toxic to living organisms causing tissue damage, disruption of the intestinal microbiota and cancer. However, there is little information on the relationship between the Cr (VI) and broiler chickens. The current study was performed to investigate the effect of Cr (VI) on growth performance, serum biochemical analysis, histopathological observations, and metabolomics analysis in broilers. Results show that Cr (VI) exposure significantly decreased the body weight (*p* < 0.01) and caused liver damages in broilers. With the extension of Cr (VI) action time, the liver appeared obvious pathological changes, including hepatic cord disorder, incomplete hepatocyte additionally, decreased serum biochemical indices of calcium (Ca), phosphorus (P), total protein (TP), phosphatase (ALP), and globin (GLB) significantly (*p* < 0.01). Moreover, metabolomics analysis indicated that 29 differential metabolites were identified, such as phytosphingosine, L-Serine, 12, 13-DHOME, Alpha-dimorphecolic acid, L-Methionine, L-Phenylalanine, 3-Dehydroshikimate, L-Tyrosine, and N-Acetyl-L-phenylalanine were significantly decreased under the action of Cr (VI) (*p* < 0.05). These 29 differential metabolites are mainly involved in 35 metabolic pathways, such as aminoacyl-tRNA biosynthesis, phenylalanine metabolism, sphingolipid, and linoleic metabolism. The study revealed that exposure to Cr (VI) resulted in a decrease in growth performance and metabolism, with the hazards and toxicity in broiler chicken. The findings provided new insight and a comprehensive understanding of the relationship between Cr (VI) and broiler chickens.

## 1. Introduction

Environmental pollution is aggravated by industrial development and urban expansion, which is accompanied by the production of large amounts of pollutants, including liquid waste, exhaust gases, and other industrial waste. Heavy metals are regarded as one of the sources of pollution in waste and wastewater (1). The deposition and proliferation of heavy metals have seriously affected the ecosystem (1, 2). Chromium is the most abundant and natural element present in rocks, soil and living things (3). Chromium has various oxidation states from −2 to +6 (3), of which Cr (VI) is widely used in industrial production such as pigments, metallurgy, plastics, and chromite. Also, Cr (VI) is often found in industrial waste, sewage, combustion exhaust gas and cigarette smoke (4). Living organisms are exposed to Cr (VI) through a variety of routes, such as skin contact, food intake, and inhalation of chromium particles (5). Therefore, Cr (VI) exposure to humans or animals is very common and widespread as it is widely present in the environment and has many exposure pathways.

Cr (VI) is considered as living body carcinogens of group 1 by the International Agency for Research on Cancer (IARC) due to its involvement in DNA damage and oxidative stress (6). Cr (VI) is usually dissolved in water in the form of oxidation, migrates and accumulates in the food chain, showing strong toxicity and carcinogenicity (7). Previous studies have shown that Cr (VI) can change gene expression and induce the production of a large number of free radicals, however, the body is unable to effectively remove accumulated free radicals in a timely manner and is in a state of oxidative stress (8, 9). Currently, sufficient evidence demonstrated that Cr (VI) is associated with diseases in humans or animals (10), for example, Cr (VI) exposure to women resulted in breast cancer (3). Simultaneously, Cr (VI) contamination also affects aquatic systems and products, and in Ni’s study, hexavalent chromium caused severe toxic effects on the liver of marine medaka (11).

Global broiler chicken production has continued to grow in recent years based on demand for protein and nutrients. Statistically, global broiler chicken production reached 101.08 million tons in 2022, according to the US Department of Agriculture (USDA). Simultaneously, China’s annual broiler production reached 1.43 million tons in 2022, accounting for 14.14 percent of global broiler production and ranking in the top two in the world, according to the National Bureau of Statistics of China. Broiler chickens, especially those raised on farms near the chemical and chromite industries, are easily exposed to chromium by drinking polluted water, eating contaminated crops, or inhaling air containing chromium (12). Previous studies have found that chronic exposure to Cr (VI) leads to pronounced changes in the diversity and composition of gut microbiota in broilers, with a significant reduction in short-chain fatty acid-producing bacteria, posing a risk to broiler farming. (5). In addition, studies have shown that Cr (VI) not only causes economic losses but also poses a serious threat to public health and food safety (13). Consequently, an in-depth understanding of the negative effects of Cr (VI) on broilers is urgent and meaningful.

Metabolomic analysis has been an effective tool for visualizing the state of health and revealing endogenous metabolites (10). However, to our knowledge, few studies have been conducted to investigate the effects of Cr (VI) on broilers from a metabolic point of view. Therefore, in the study, we also focused on untargeted liquid chromatography-mass spectrometry (LC–MS) metabolic analysis to determine the changes in metabolites and health status of broiler chickens under the exposure of Cr (VI).

Collectively, in the study, we aimed to investigate the effects of Cr (VI) on broiler chickens from a new perspective. Our findings shed light on the relationship between Cr (VI) and broilers based on a series of experiments, and provides a basis for further understanding of Cr (VI). At the same time, this study further strengthens environmental risk assessment and protection.

## 2. Materials and methods

### 2.1. Animal experiment

In total, 60 broiler chickens (1 day old, 45–53 g) were bought from a commercial hatchery (Jingzhou, China) and transported to the Huazhong Agricultural University Animal Centre. All broilers were housed in the standard environmental conditions (temperature, 33 ± 3°C; humidity, 55 ± 10%) and acclimatized to the environment for 7 days prior to the experiment. At the beginning of the experiment, to ensure the safety and accuracy of the research, 60 broiler chickens were randomized to two groups: Group A (*n* = 30) and group B (*n* = 30). Broilers in group A were provided with a standard diet, and broilers in group B were fed the standard diet supplemented with potassium dichromate (K_2_Cr_2_O_7_). According to Li’s method (5), K_2_Cr_2_O_7_ was dissolved in water and K_2_Cr_2_O_7_ was fed at the supplemental level of 0.07424 mg/kg/d according to the body weight of broilers for 35 days.

### 2.2. Body weight measurement and serum biochemical analysis

The body weight of the broilers was measured once a week during the experiment, i.e., initial body weight, day 7, day 14, day 21, day 28, and day 35, respectively. In addition, blood was taken from the jugular vein of broiler chickens by means of a 21 gauge needle on day 21 and day 35, respectively. The serum was separated after centrifuging (1,000 g/min, 20 min), and 200 mL of serum was transferred to a new tube for serum biochemistry analysis. The biochemical assays included calcium (Ca), phosphorous (P), total protein (TP), total cholesterol (TC), alkaline phosphatase (ALP), alanine aminotransferase (ALT), globin (GLB), and aspartate aminotransferase (AST). All serum biochemical indexes were measured using biochemical assay kits (Nanjing Jianjian Bioengineering Research Institute, Nanjing, China) according to the manufacturer’s operating procedures.

### 2.3. Histological pathological observations

At the end of the experiment (on day 35), all broiler chickens were humanely slaughtered after 24 h of fasting according to Li’s method (5). The liver from each broiler in both groups were fixed in 4% paraformaldehyde for 48 h, followed by dehydration, cleaning and embedding. Finally, the tissue sections were stained with hematoxylin and eosin (H&E) staining (14). The histopathological changes in tissue sections were observed under an inverted microscope.

### 2.4. Pretreatment and metabolomics profiling of serum samples

Serum samples were thawed at 4°C and vortexed for 1 min. Accurate transfer of 100 μL sample into a 2 mL centrifuge tube for metabolites separation and extraction (<1,500 Da). First, 400 μL of methanol was added to vortex with the samples for 1 min and centrifuged at 12000 rpm for 10 min at 4°C. The supernatant was then transferred to a new 2 mL centrifugal tube and concentrated. After that, 150 μL 2-chloro-I-phenylalanine solution was dissolved with the sample. At last, the supernatants were filtered by a membrane (0.22 mm) and transferred into the decision bottle for liquid chromatography–tandem mass spectrometry (LC–MS) detection.

The ACQUITY UPLC System (Waters, Milford, MA, United States) coupled to a LTQ Orbitrap XL instrument (Thermo Fisher Scientific, United States). Simultaneous sample analysis was performed by MS1 and MS/MS (Full MS-ddMS2 mode, data-dependent MS/MS) acquisition. The parameters were set as follows: spray voltage of 45 carbs, aux gas flow of 15 carbs, a capillary temperature of 325°C, spray voltage, 4.80 kV and −4.50 kV for ESI(+) and ESI (−), respectively. MS1 range, m/z 89–1,000 and 114–1,000 in the positive and negative modes, respectively. The LC–MS system was performed by reference to Zelena’s method (15). The mobile phases consisted of A and B, of which solvent A was 0.1% formic acid in water (v/v) and solvent B was 0.1% formic acid in acetonitrile (v/v). The separation gradient was set as follows: 2% B at 0–1 min, 10% B at 5 min, 98% B at 8 min, 98% B at 10 min, 2% B at 15 min, and 2% B at 16 min. Additionally, the flow rate was set to 0.25 mL/min and injection volume was set 5 μL.

The acquired LC–MS raw data were converted to mzXML format by MSConvert using Proteowizard software (v3.0.8789). The raw data were then processed using R XCMS software, including peak selection, integration and retention correction. Finally, to further explore differences in metabolic profiles, the processed data were subjected to principal component analysis (PCA), partial least squares discriminant analysis (PLS-DA), and orthogonal partial least squares discriminant analysis (OPLS-DA). Metabolites with significant differences were identified based on variable importance plot (VIP) > 1 and *p* < 0.05. Concurrently, correlations between metabolites were analysed by calculating the Pearson correlation coefficient. In addition, metabolic pathways of different metabolites were determined by MetaboAnalyst and the KEGG database.

### 2.5. Statistical analysis

In the study, data were analyzed by one-way analysis of variance (ANOVA) through SPSS (v 23.0) and box plots were drawn by Graphpad Prism software (v 8.0). Also, data were expressed as means + SD, and the criterion for determining a statistically significant difference was *p* < 0.05.

## 3. Results

### 3.1. Growth performance

**Figure 1:**
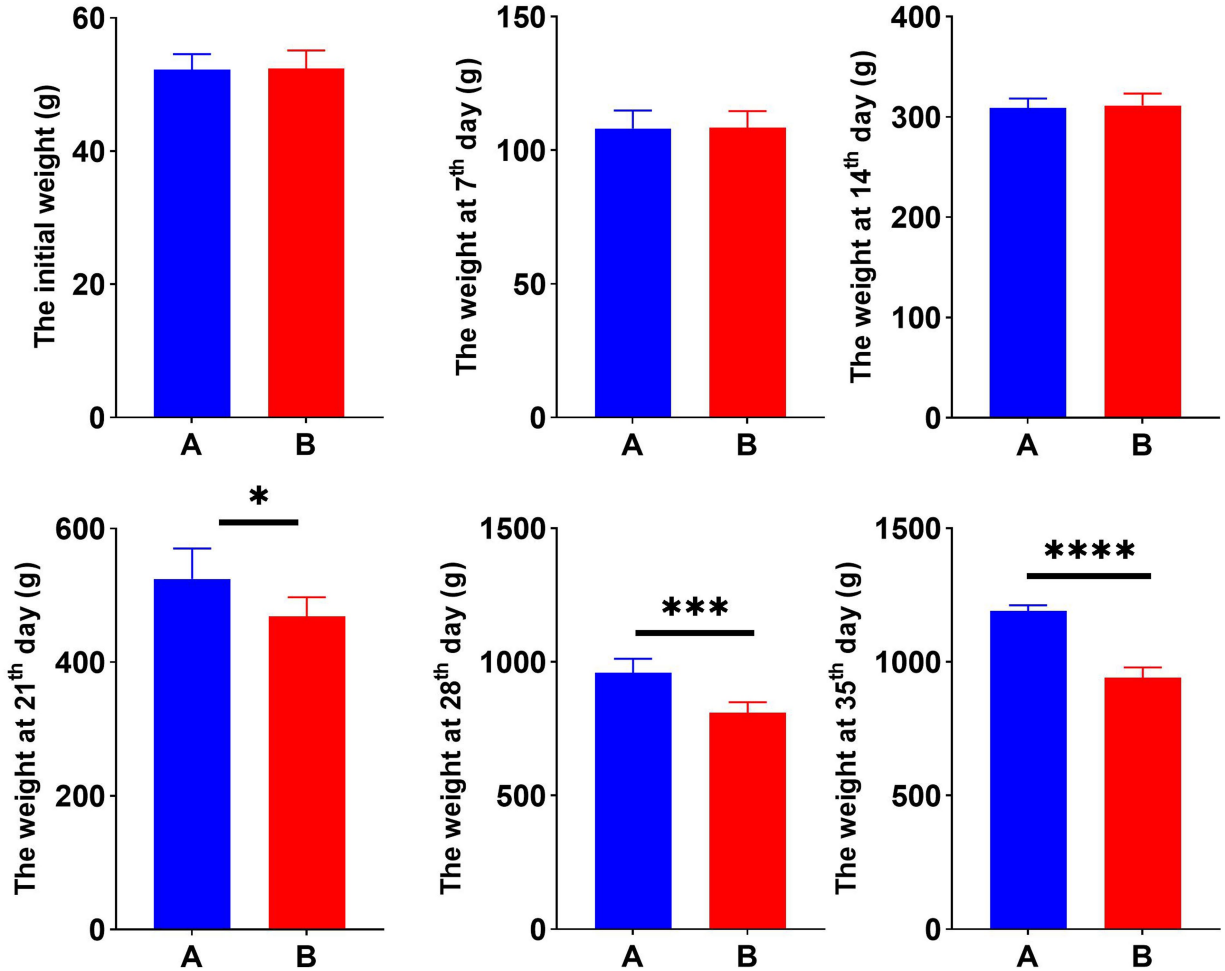
Hexavalent chromium (Cr VI) decreased the body weight of broilers. Dynamic changes in body weight of broilers in groups A (standard diet) and B (standard diet and chromium), i.e., initial weight and weekly post-trial weight records. *p < 0.05, ***p < 0.01, and ****p < 0.001.

To examine the effect of Cr (VI) on growth performance in broilers, we constructed a Cr (VI)-infection model by feeding broilers with Cr (VI). Our findings showed no significant difference between the two groups during the first 14 days of the study. At day 21, however, there was a significant difference in body weight between groups A and B. Moreover, the difference became more pronounced as the day progressed. As shown in Figure 1, at day 35, the average body weight of broilers in the B group was significantly lower than that of the A group, with a different value of approximately 250.8 g (*p* < 0.01) (Figure 1).

### 3.2. Histological observation of the liver

**Figure 2:**
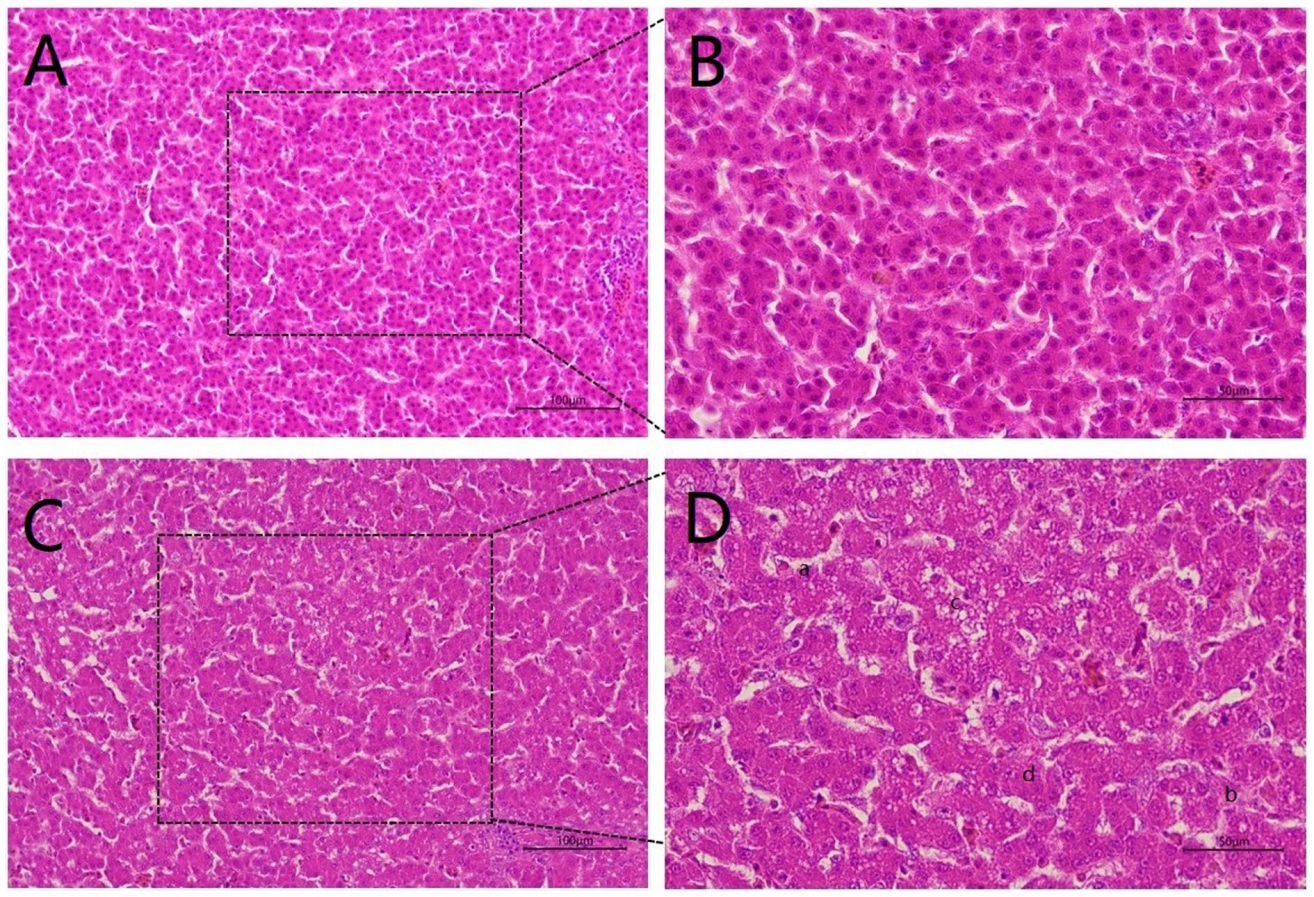
Hexavalent chromium (Cr VI) caused liver pathological changes. (**A, B**) The H&E staining of liver histology in the control group. There was no damage to the liver, which was structurally intact and displayed a healthy state with neat and regular liver cells. **(A)**: 20X microscope observation. **(B)**: 40X microscope observation. (**C, D**) Liver histological changes of broilers in the B group (Cr (VI) + standard diet). **(C)**: 20X microscope observation. **(D)**: 40X microscope observation. Cr (VI) exposure causes serious liver damage, which is summarized as follows. a. Disordered hepatic cord arrangement; b. Hepatocytes rupture; c. Vacuoles are found in the cytoplasm; d. Absence of nucleus.

To explore the hazards of Cr (VI) on broilers, we observed histopathological changes in the liver. As shown in Figure 2, the liver structure of broilers fed a standard diet exhibits a healthy state, with liver cells being neat and regular (Figures 2A,B). In turn, the livers of broiler chickens in the Cr group exhibit obvious pathological changes, with disturbed hepatic cords and incomplete hepatocytes, accompanied by rupture and lysis of the hepatocytes. In addition, vacuoles were found in the cytoplasm and cell nuclei were lost, which was suspected to be due to steatosis (Figures 2C,D).

### 3.3. Serum biochemistry test

**Figure 3:**
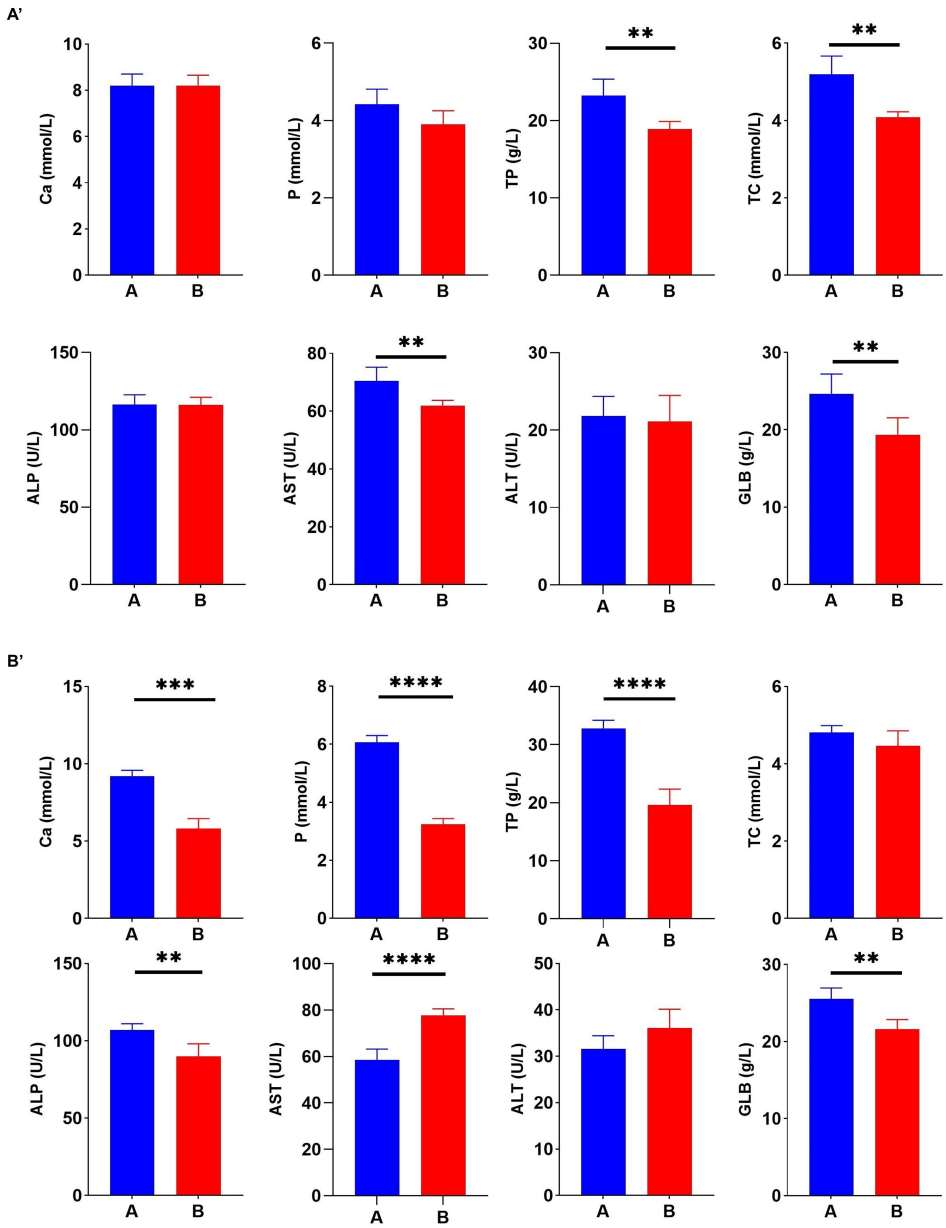
Hexavalent chromium (Cr (VI)) exposure altered the serum biochemical index of broilers. **(A′)** Serum biochemical indices on day 21. **(B′)** Serum biochemical indices on day 35. **(A)** The control group. **(B)** Cr (VI)-exposure group.

To better understand the correlation between Cr (VI) and broiler, serum biochemical tests were performed during the study period, at days 21 and 35, respectively. On day 21, TP, TC, AST, and GLB levels in Group A were significantly higher than those in the B group (*p* < 0.01) (Figure 3A). As expected, this difference became more significant as the duration of the experiment increased. On day 35, Cr (VI) significantly decreased Ca, P, TP, ALP, and GLB levels of broilers (*p* < 0.01), and conversely, AST levels increased (*p* < 0.01) (Figure 3B).

### 3.4. The effect of Cr (VI) on the metabolism of the body in broilers

**Figure 4:**
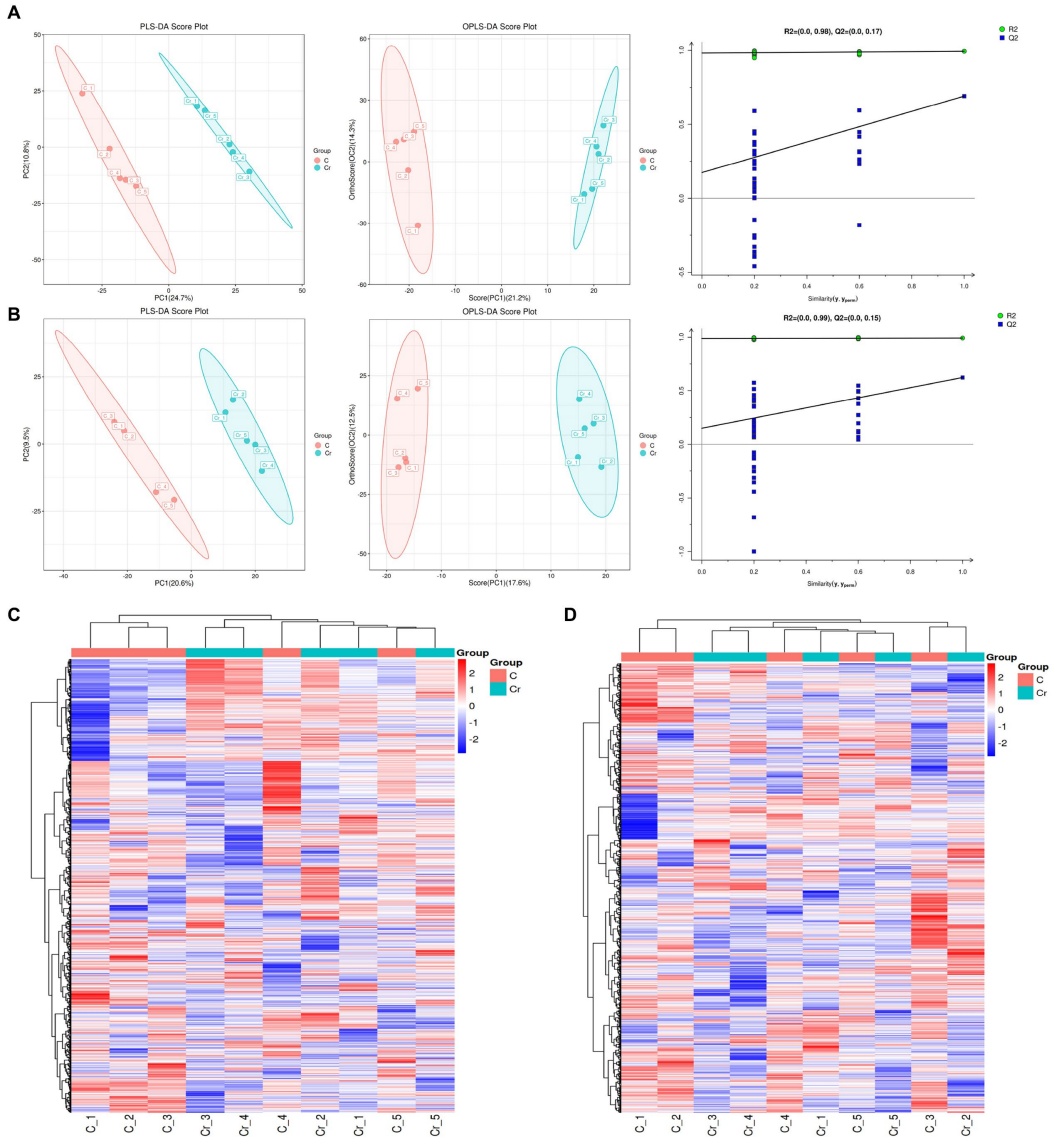
The metabolites analysis between groups A (C stands for Group A) and B (Cr stands for Group B). **(A)** In the positive model, multivariate statistical analyses including PLS-DA score spot and OPLS-DA score spot showed the difference of metabolites between groups. Additionally, the permutation test of OPLS-DA demonstrated the reality and reliability of the OPLS-DA model. **(B)** PLS-DA score spot, OPLS-DA score spot, and permutation test of OPLS-DA in the negative mode. **(C)** The heatmap of metabolites in the positive model. **(D)** The heatmap of metabolites in the positive model.

To further explore the correlation between Cr (VI) and health in broilers, we performed non-target metabolic profiling of serum in both groups. PLS-DA and OPLS-DA in the positive and negative modes showed that the dots in the B group were clearly separated from those of group A, indicating significant differences in serum metabolism between groups A and B (Figures 4A,B). Additionally, the permutation test of OPLS-DA showed that values of R2 and Q2 were both lower than original of R2 and Q2 on the upper right, which demonstrated the validity and authenticity of the OPLS-DA model (Figures 4A,B).

As depicted in Figures 4C,D, 133 metabolites were found in both groups. Alpha-dimorphecolic acid, Aminoadipic acid, 4-Hydroxycinnamic acid, and N-Acetyl-L-phenylalanine (VIP > 1).

### 3.5. The effect of Cr (VI) on the metabolism pathways in broilers

**Figure 5:**
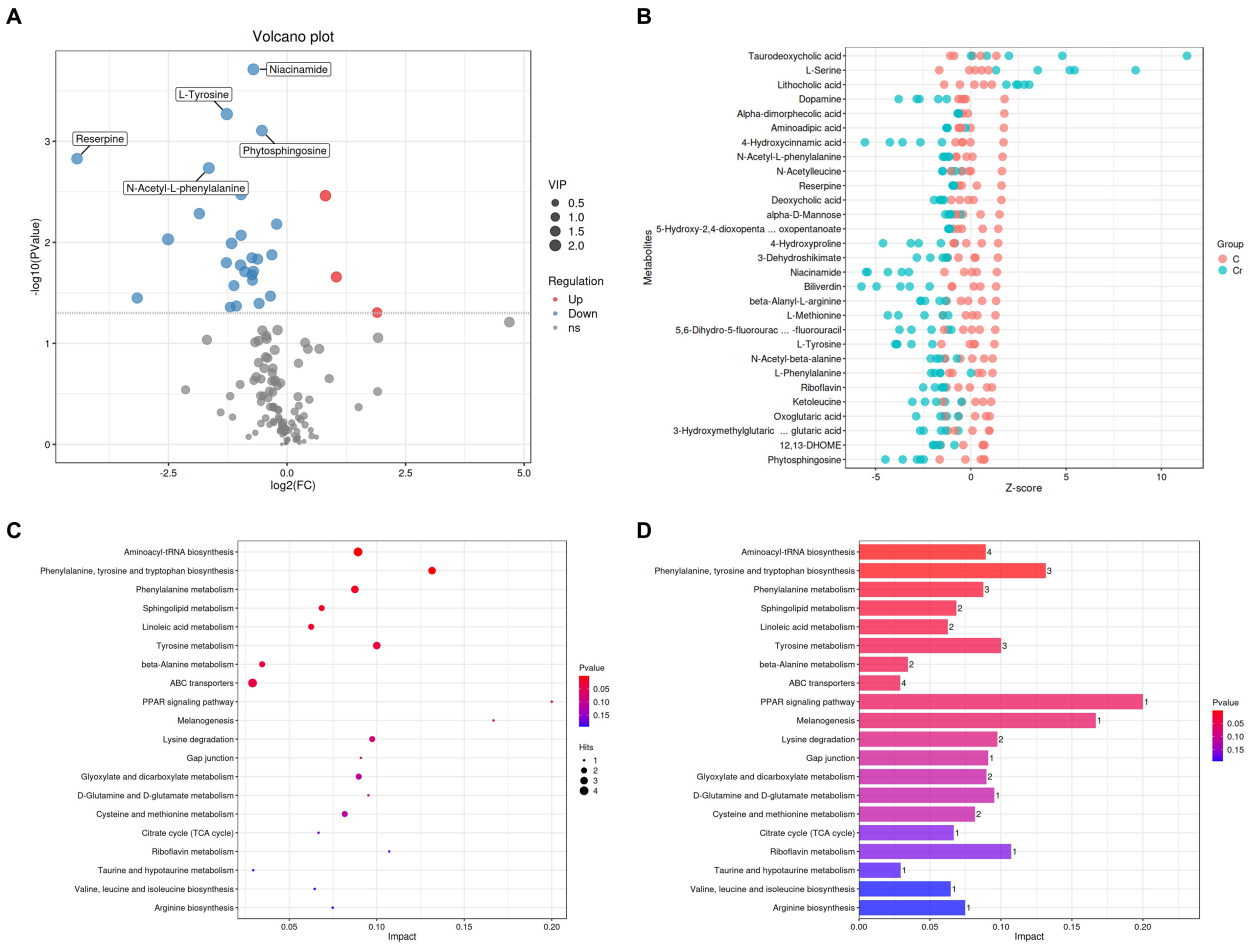
Cr (VI) exposure significantly altered metabolite concentrations and metabolic pathways in broilers. **(A)** Volcano plots manifested the differential metabolites. The red colored dots indicate the increased concentration of metabolites in the Cr (VI)-exposure group compared to the control group. In turn, the blue dots indicate a decrease in the concentration of metabolites under Cr (VI) effect compared to the control group. **(B)** Metabolite content (*Z*-score indicate the content) in groups A (C stands for Group A) and B (Cr stands for Group B). Closer to the right, the relative content of the current metabolite in that sample is higher, and closer to the left, the current metabolite content is lower. (**C, D**) Enrichment analysis of metabolic pathways.

**Figure 6:**
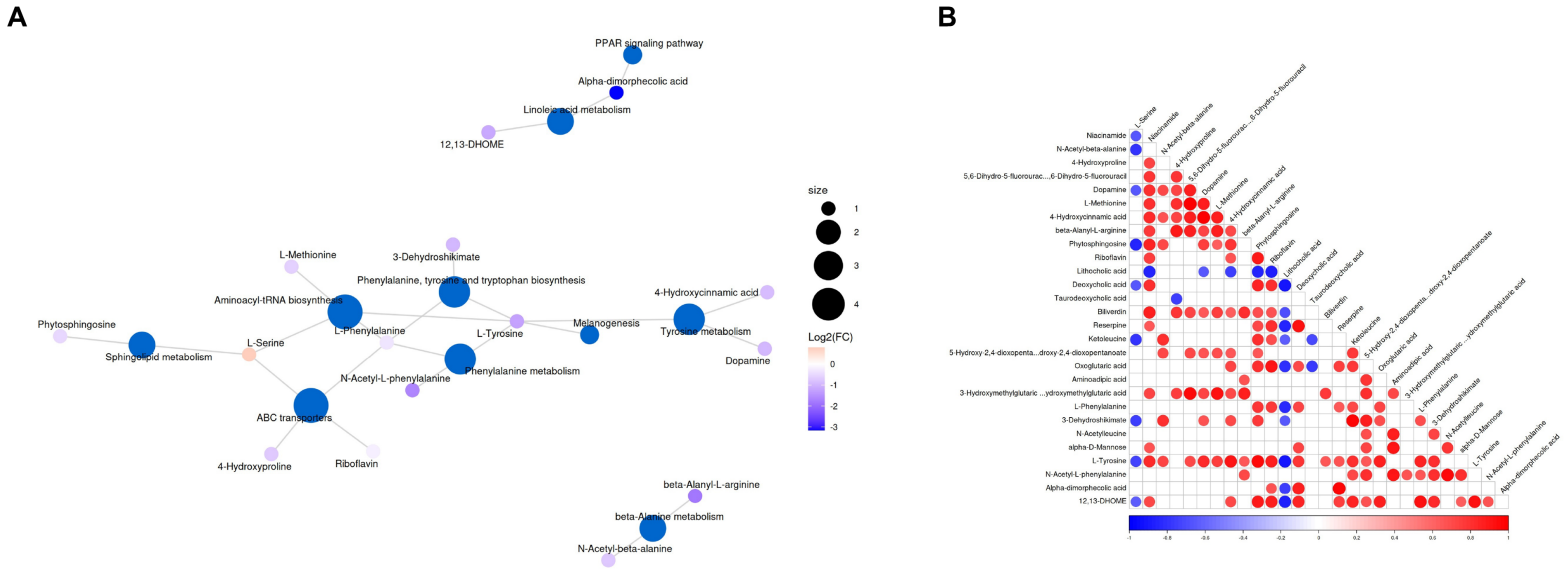
**(A)** Top representative metabolism pathways with extremely significant impact value between groups A and B. **(B)** Correlation analysis of differential metabolites. Red and blue indicate positive and negative correlations, respectively, *p* < 0.05 represents significant correlation.

The significantly different metabolites were subjected to KEGG pathways analysis using MetaboAnalyst^1^. The data indicated that significantly different metabolites were involved in 35 pathways, and the representative pathways were shown in Figures 5C,D. Among the representative pathways, the top 5 pathways with extremely significant impact value were aminoacyl-tRNA biosynthesis, phenylalanine tyrosine and tryptophan biosynthesis, phenylalanine metabolism, sphingolipid metabolism and linoleic metabolism. As shown in the metabolic diagram, 9 potential biomarkers were associated with the top 5 pathways, including phytosphingosine, L-Serine, 12, 13-DHOME, Alpha-dimorphecolic acid, L-Methionine, L-Phenylalanine, 3-Dehydroshikimate, L-Tyrosine, N-Acetyl-L-phenylalanine (Figure 6A).

### 3.6. The correlations between different metabolites

Correlation analysis was conducted by calculating Pearson’s correlation coefficient to detect the associations between differential metabolites. In our findings, niacinamide was negatively associated with L-Serine and N-Acetyl-beta-alanine. 4-Hydroxyproline was positively associated with N-Acetyl-beta-alanine. Dopamine was negatively associated with L-Serine but positively associated with Niacinamide, N-Acetyl-beta-alanine, 4-Hydroxyproline and 5, 6-Dihydro-5-fluorourac. L-Methionine was positively associated with Niacinamide, 4-Hydroxyproline, and Dopamine. 4-Hydroxycinnamic acid was positively associated with Niacinamide, N-Acetyl-beta-alanine, 4-Hydroxyproline, 5, 6-Dihydro-5-fluorourac, Dopamine and L-Methionine. Beta-Alanyl-L-arginine was positively associated with Niacinamide, 4-Hydroxyproline, 5, 6-Dihydro-5-fluorourac, Dopamine, L-Methionine and 4-Hydroxycinnamic acid. Phytosphingosine was negatively associated with L-Serine, but positively associated with Niacinamide, N-Acetyl-beta-alanine, Dopamine, L-Methionine and 4-Hydroxycinnamic acid (Figure 6B).

## 4. Discussion

Heavy metal contamination in the environment (such as air, soil, and groundwater) has been a serious concern for animal husbandry and food safety, which affect the living organisms health by perturbing gut microbiota or metabolism (16). Hexavalent chromium is considered a hazardous metal contaminant, and there is increasing evidence that hexavalent chromium can cause cancer, tumor development, gastrointestinal disorders, and liver damage (3, 16, 17). The Cr (VI) promotes the formation of active oxygen forms (AFCs), followed by cellular damage (3, 18). Zhitkovich et al. demonstrated that Cr (VI) can interact with amino acids or DNA, resulting in DNA breakage or damage (19). However, information regarding the correlation between Cr (VI) and broilers is limited, especially from the growth and metabolism perspective. Metabolism, the sum of chemical reactions and energy absorption and utilization processes, and the synthesis of organic matter. Therefore, the correlation of Cr (VI) and broilers from the metabolism point is necessary and urgent.

In the study, the hazards of Cr (VI) on the growth performance of broilers was notable. As the data shows, Cr (VI) significantly reduced the body weight of broilers compared to the control group, and the difference becomes more pronounced with the extension of time. Our data are consistent with Zhang’s study, where Cr (VI) resulted in significant weight loss in mice (16). Based on previous research (16, 20) and our data, a possible explanation is that Cr (VI) may disrupt the gut microbiota balance and body metabolism, resulting in weight loss. Our data have provided evidence that calcium (Ca) and phosphorus (P) levels have been significantly reduced (*p* < 0.05) in the B group compared to the control group, indicating Cr (VI) impair the metabolism of Ca and P. Similarly, Brzoska et al. also demonstrated that cadmium retention and accumulation has severe negative effect on calcium metabolism in rats (21). Alanine aminotransferase (ALT) and aspartate aminotransferase (AST) are important circulating enzymes, indicating the damage and death of liver cell (22). The new study also suggests that ALT and AST should be considered as significant indicators for predicting malignant tumour. For example, in the study of Zhou et al., the AST/ALT ratio is a reliable predictor for estimating the risk of prostate cancer incidence (23). Meaningfully, we observed that the level of AST in the A group was significantly higher than that in the B group (*p* < 0.01), indicating that Cr (VI) had a damaging effect on the liver of broilers. Simultaneously, we found that chromium reduced the levels of TP and GLB, which may be related to the hepatotoxic effects of hexavalent chromium. Our findings are consistent with the report by Rosa et al. The damaged liver cells are characterized by inhibition of protein synthesis accompanied by a decrease in TP and GLB (24). On the other hand, the liver of a broiler exposed to Cr (VI) for an extended period of time showed distinct pathological changes, including hepatic cord disorders, hepatocyte rupture, and vacuoles in the cytoplasm. Our findings are consistent with Ni’s report that Cr (VI) also causes acute pathological changes in the liver of marine medaka, including cellular vacuolation, nuclear aggregation, and migration (11).

Metabolites can serve the function by acting on organs, including stomach, kidney, liver, genital system, and blood circulation, therefore metabolism acts as an important role in living organisms, determining the health status of living organisms throughout life. In our finding, it was shown that a total of 29 metabolites were affected by hexavalent chromium, including 3 upregulated and 26 downregulated metabolites. Then, metabolites with significantly different profiles (*p* < 0.05) were subjected to KEGG pathway analysis. Cr (VI) exposure clearly affects 35 metabolic pathways, of which 5 representative pathways are aminoacyl-tRNA biosynthesis, phenylalanine, tyrosine and tryptophan biosynthesis, phenylalanine metabolism, sphingolipid and linoleic metabolism. The altered metabolites and metabolic pathways may be the main cause of the negative effects of Cr (VI) on the host. In the study, we found significant changes in the Aminoacyl-tRNAs synthesis pathway. Previous researches have demonstrated that aminoacyl-tRNAs synthesis pathway plays a crucial role in matching amino acids with tRNAs containing the corresponding anticodon, precisely identifying the genetic code to be interpreted as the corresponding amino acid (25–27). One possible explanation for this change is that Cr (VI) impairs the synthesis of aminoacyl-tRNAs. Next, a significant change was observed in phenylalanine, tyrosine and tryptophan biosynthesis pathway, accompanied by the significant reduction in N-Acetyl-L-phenylalanine, L-phenylalanine and L-Tyrosine (*p* < 0.05). Amino acids are essential for the maintenance and promotion of metabolism, including immune regulation, oxidative stress regulation and protein biosynthesis (28–30), of which L-Tyrosine is a valuable amino acid with a variety of applications, contributing to stimulating brain activity, enhancing brain memory and mental alertness, inhibiting depression, relieving the pressure, and promoting growth performance (31). What’s more, L-Tyrosine is a precursor to the synthesis of catecholamine, dopamine (DA), and norepinephrine (NE) (32). Simultaneously, Cr (VI) also caused changes in phenylalanine metabolism together with significant reduce of phenylalanine level. Among the latest points, the metabolism of phenylalanine is closely linked to the disease, for example, in Liu’s report, the metabolism of phenylalanine is dysregulated in Alzheimer’s disease (33). Phenylalanine is an important aromatic amino acid that acts as a precursor to most essential and vital building blocks of living organisms (34, 35). Numerous studies claimed that phenylalanine is commonly associated with glycose metabolism and lipid metabolism, and is involved in regulating homeostasis and health (36–40). Therefore, we speculate that Cr (VI) exposure affects glucose metabolism and lipid metabolism by impairing phenylalanine metabolism and decreasing phenylalanine levels. This may be the reason why Cr (VI) exposure reduces body weight in broilers.

Interestingly, our results demonstrate that Cr (VI) changed the sphingolipid and linolenic acid metabolism in broilers, which confirmed our hypothesis. Linolenic acid metabolism is an important metabolism pathway involved in maintaining health and regulating the immune system (41–43). In the study of Zhang et al., they pointed out that the disturbance of linoleic acid metabolism may cause cardiovascular disease (44). Similarly, Wang et al. also demonstrated that linoleic acid metabolism is strongly associated with chronic heart failure (45). Also, Cr (VI) significantly reduced the levels of short chain fatty acid (SCFAs) (*p* < 0.05), including Alpha-dimorphecolic acid, aminoadipic acid, 4-hydroxycinnamic acid, and deoxycholic acid. Increasing evidence demonstrated that linolenic acids are vital and essential monounsaturated fatty acids which contribute to maintain heart health and prevent disease (such as cardiovascular) (44, 46–49). Significantly, Cr (VI) exposure also reduced the level of nicotinamide in broilers (*p* < 0.05). Previous research has shown that niacinamide is an important amide of vitamin B3 (niacin), which has functions of anti-pruritic, vasoactive, antimicrobial, photoprotective, sebostatic and brighten effects in living organisms. Additionally, niacinamide is able to inhibit the nuclear poly (ADP-ribose) polymerase-1 (PARP-1) and controls the NFκB-mediated transcription of signalling molecules (7, 50). It is worth noting that there is a correlation between the reduction of niacinamide and the addition amount of Cr (VI), which is likely to be the reason for the poor growth performance of broilers in Cr group.

## 5. Conclusion

Overall, our study revealed complex changes in broiler chicken exposure to Cr (VI) with respect to growth and metabolism. Cr (VI) has considerably reduced the growth of broiler chickens, characterized by weight loss and liver damage. Additionally, the serum metabolism results highlight the toxic effect and potential hazards of Cr (VI) exposure to broilers, including 35 metabolic pathways and 29 metabolites biomarkers were dramatically altered. The changed metabolic pathways were essential and crucial to the health of living organisms, such as aminoacyl-tRNA biosynthesis, phenylalanine metabolism, this study not only broadens the understanding of the hazards and risks of Cr (VI), but also reveals that metabolic alterations may be a key pathway for Cr (VI) to exert its toxic effects. Meaningfully, we also found a correlation between some metabolites. These differently metabolites may affect the other metabolite level through the interaction under the action of Cr (VI).

## Data availability statement

The raw data supporting the conclusions of this article will be made available by the authors, without undue reservation.

## Ethics statement

The animal study was approved by Ethics Committee of Huazhong Agricultural University. The study was conducted in accordance with the local legislation and institutional requirements.

## Author contributions

GZ: writing – original draft and experiment design, supervision, and funding acquisition. LW: experiment. LW, MA, and MH: writing – review and editing. ZJ: investigation.

## Footnotes

^1^ www.metaboanalyst.ca
